# Draft Genome Sequence of Marine *Bacillus* sp. Strain ISO11, a Candidate Finfish and Shellfish Probiotic

**DOI:** 10.1128/MRA.01227-18

**Published:** 2018-10-18

**Authors:** Harold J. Schreier

**Affiliations:** a Department of Marine Biotechnology, Institute of Marine and Environmental Technology, University of Maryland Baltimore County, Baltimore, Maryland, USA; b Department of Biological Sciences, University of Maryland Baltimore County, Baltimore, Maryland, USA; Louisiana State University

## Abstract

Bacillus sp. strain ISO11, a Bacillus cereus clade member isolated from the intestinal tract of Fundulus heteroclitus, possesses potential probiotic and antibacterial activity against Vibrio sp. pathogens. Antibacterial activity is likely due to production of microcin and a zwittermicin A-like aminopolyol. The genome sequence will assist in identifying additional related processes.

## Abstract (precis)

Bacillus sp. strain ISO11, a Bacillus cereus clade member isolated from the intestinal tract of Fundulus heteroclitus, possesses potential probiotic and antibacterial activity against Vibrio sp.

## ANNOUNCEMENT

The use of probiotic bacteria rather than chemical treatments, such as disinfectants or antibiotics, has become a strategy for controlling the proliferation of microbial pathogens in aquaculture (1). Through a study aimed at identifying marine bacteria exhibiting probiotic potential, Bacillus sp. strain ISO11 was isolated from the intestine of an adult Fundulus heteroclitus and was examined for antibacterial and probiotic activities. Bacillus spp. possessing probiotic activity are of interest to the aquaculture industry due to the long-term shelf life and chemical resistance of their spores (2). The genome sequence of Bacillus sp. ISO11 will facilitate an understanding of processes involved in its antibacterial and probiotic activities.

A single colony was grown in marine broth 2216 (Difco) at 28°C, and DNA was extracted using the Wizard genomic DNA purification kit (Promega). The genome library was prepared using Illumina Nextera XT chemistry, and sequencing was done with an Illumina MiSeq benchtop sequencer. The read library contained 8,701,622 paired-end reads with an average read length of 250 bp and average coverage of 367×. *De novo* assembly was done using the Pathosystems Resource Integration Center (PATRIC v 3.5.22) (3) genome assembly resource with the MiSeq parameter, yielding 107 contigs consisting of 5,924,228 bp total, with an *N*_50_ of 150,352 bp and GC composition of 34.9%. Gene prediction and annotation using the Rapid Annotation using Subsystem Technology tool kit (RASTtk) (4) generated 6,298 coding sequences. RASTtk 16S rRNA phylogenetic analysis placed ISO11 in the Bacillus cereus clade, with the greatest similarity to Bacillus cereus.

Genome sequence analysis by BLAST (5) revealed approximately 86 kb of DNA encoding polyketide synthases and related proteins having high (>98%) similarity to the aminopolyol zwittermicin A cluster of several B. cereus strains, including UW85 (6). Zwittermicin A is active against Gram-negative and Gram-positive bacteria as well as fungi and protists (7). Differences in deduced polyketide synthase amino acid sequences suggest that ISO11 produces an aminopolyol similar but not identical to zwittermicin A. Like UW85, ISO11 appears to lack *zmaWXY* genes associated with self-resistance found in the zwittermicin A cluster of Bacillus thuringiensis YBT-1520 (8). Genes for biosynthesis of a thiazole/oxazole-modified microcin (TOMM) antimicrobial agent that may play a role in probiotic activity (9), along with an associated microcin C7 immunity-encoding *mccF*, were also identified.

The ISO11 genome encodes motility, adhesion, and aggregation functions needed for probiotic activity, including flagella (10), exopolysaccharide biosynthesis (*epsBCDE* genes) (11), enolase (12), and fibronectin (13), as well as arginine deiminase pathway enzymes for protection against the acidic environment of the host’s stomach (14).

Hemolysin and hemolysin-like proteins are encoded, including hemolysin BL (Hbl) and nonhemolytic enterotoxin (Nhe), as well as cytotoxin K (CytK), which are causative agents of gastrointestinal disease (15). While ISO11 has not been shown to cause disease in finfish or shellfish (H. J. Schreier, E. J. Schott, and D. McIntosh, unpublished data), the genome sequence will be advantageous for characterizing ISO11-host interactions.

### Data availability.

This whole-genome shotgun project has been deposited at DDBJ/ENA/GenBank under the accession number QXCO00000000. The version described in this paper is the first version, QXCO01000000. Sequence data have been deposited in the Sequence Read Archive under the accession number SRP162544.
